# Lingual Muscle Activity Across Sleep–Wake States in Rats with Surgically Altered Upper Airway

**DOI:** 10.3389/fneur.2014.00061

**Published:** 2014-04-28

**Authors:** Irma Rukhadze, Julie Kalter, Georg M. Stettner, Leszek Kubin

**Affiliations:** ^1^Department of Animal Biology, School of Veterinary Medicine, University of Pennsylvania, Philadelphia, PA, USA

**Keywords:** genioglossus, hyoid bone, obstructive sleep apnea, REM sleep, tongue

## Abstract

Obstructive sleep apnea (OSA) patients have increased upper airway muscle activity, including such lingual muscles as the genioglossus (GG), geniohyoid (GH), and hyoglossus (HG). This adaptation partially protects their upper airway against obstructions. Rodents are used to study the central neural control of sleep and breathing but they do not naturally exhibit OSA. We investigated whether, in chronically instrumented, behaving rats, disconnecting the GH and HG muscles from the hyoid (H) apparatus would result in a compensatory increase of other upper airway muscle activity (electromyogram, EMG) and/or other signs of upper airway instability. We first determined that, in intact rats, lingual (GG and intrinsic) muscles maintained stable activity levels when quantified based on 2 h-long recordings conducted on days 6 through 22 after instrumentation. We then studied five rats in which the tendons connecting the GH and HG muscles to the H apparatus were experimentally severed. When quantified across all recording days, lingual EMG during slow-wave sleep (SWS) was modestly but significantly increased in rats with surgically altered upper airway [8.6 ± 0.7% (SE) vs. 6.1 ± 0.7% of the mean during wakefulness; *p* = 0.012]. Respiratory modulation of lingual EMG occurred mainly during SWS and was similarly infrequent in both groups, and the incidence of sighs and central apneas also was similar. Thus, a weakened action of selected lingual muscles did not produce sleep-disordered breathing but resulted in a relatively elevated activity in other lingual muscles during SWS. These results encourage more extensive surgical manipulations with the aim to obtain a rodent model with collapsible upper airway.

## Introduction

Studies of upper airway control in obstructive sleep apnea syndrome (OSA) patients revealed that upper airway narrowing and collapse during sleep is caused by decrements of activity in upper airway dilator muscles (1–3). Studies also have demonstrated that OSA patients have elevated upper airway motor tone during wakefulness, which allows them to maintain adequate ventilation (4–7). However, this compensatory increase is diminished during sleep, making the airway vulnerable to collapse. OSA affects at least 5% of adults (8, 9). It leads to episodic nocturnal hypoxia, sleep loss, and sleep fragmentation which, in turn, are major contributing factors to cardiovascular and metabolic disorders, and increased mortality (10–14).

Although certain breeds of dogs and cats and rabbits under certain experimental conditions exhibit sleep-related upper airway narrowing or collapse (15–17), OSA is an almost uniquely human disorder. This is probably due to distinct features of human upper airway anatomy, including the absence of a rigid support for the hyoid (H) apparatus and elongation of the pharyngeal region in association with the development of speech. The scarcity of animal models is a challenge for the studies that attempt to capture the neuromechanical features important for upper airway control in OSA patients and investigate various consequences of sleep-disordered breathing.

Rodents are extensively used to study the central neural control of sleep and breathing, but they exhibit no propensity for spontaneous airway obstructions either awake or asleep (18–20). Thus, there is a need for a rodent model with anatomically compromised upper airway in a way that would bear similarity to the neuromechanical conditions present in OSA patients. Here, we present an attempt to develop a rodent model with experimentally weakened muscular support of the upper airway and test whether this intervention leads to increased upper airway muscle tone analogous to that seen in OSA patients.

The extrinsic muscle of the tongue, such as the genioglossus (GG), geniohyoid (GH), and hyoglossus (HG), regulate the position and stiffness of the tongue, thereby also protecting the upper airway from collapse in OSA patients (3, 21–23). In both rats and humans, GH muscle fibers run parallel to the horizontal part of the GG muscle (24, 25). Therefore, their co-activation stiffens the floor of the mouth. The human HG muscle is classified as a tongue retractor, but its other major function is to pull the dorsal surface of the tongue down. As such, a co-activation of HG together with GG and GH should further stiffen the pharyngeal walls [cf. (26)]. Thus, while GG is the main tongue protruder, GH and HG muscles can act as its synergist in fulfilment of their function to protect the pharyngeal airway from collapse. Both, GH and HG are attached on one end to the H bone. Therefore, we hypothesized that disconnecting them from the H apparatus would reduce the effectiveness of their contraction and weaken the support they provide to the GG muscle. This, in turn, could compromise upper airway patency, especially during sleep and, by increasing the load on other synergistic muscles that are left intact, such as GG, lead to a compensatory increase of their activity. In order to test this hypothesis, we quantified lingual muscle electromyogram (EMG) during wakefulness, slow-wave sleep (SWS), and rapid eye movement sleep (REMS) in control rats and rats with GH and HG muscles disconnected from the H apparatus. We determined that GG and intrinsic muscle activity in rats with GH/HG disconnected from H apparatus was moderately elevated during SWS when measured relative to its level during wakefulness. However, the incidence of transient respiratory instabilities and respiratory modulation of lingual muscle activity were similar in both groups. A preliminary report has been published (27).

## Materials and Methods

Seventeen adult, male Sprague-Dawley rats obtained from Charles River Laboratories (Wilmington, MA, USA) were used. Of those, seven animals were excluded when it was determined early after instrumentation that the quality or stability of the signals was not satisfactory (*n* = 3) or when histological analysis after completion of the study revealed that the recording sites within lingual muscles were not satisfactorily located near the base of the tongue (*n* = 4). Ten rats with satisfactory lingual recording sites and no evidence of malfunction of implanted electrodes were used for data analysis.

At the time of instrumentation, the average body weight of the 10 rats included in the study was 344 ± 17 g (standard error – SE). All animal handling and surgical procedures were approved by the Institutional Animal Care and Use Committee of the University of Pennsylvania and followed the guidelines of the American Physiological Society for the care and use of animals in research.

### Instrumentation for monitoring of sleep–wake behavior and EMG activity

The animals were pre-anesthetized with isoflurane (3–5%) followed by ketamine (60 mg/kg, i.m.) and xylazine (7.5 mg/kg, i.m.), and then by isoflurane administered through a nose mask (0.5–0.8%). They were then instrumented for recording of the cortical EEG and lingual, nuchal, and sternal diaphragmatic EMGs. For recording from lingual muscles, two 10-stranded, teflon-coated stainless steel wires (#AS636; Cooner Wire, Chatsworth, CA, USA) were implanted, one on each side, into the tongue near its pharyngeal region, as described previously (28). The recording ends of the wires had insulation removed over ~0.1 mm. In five animals that are thereafter referred to as the “experimental rats,” the position of the cartilages comprising the medial-anterior part of the H apparatus to which GH and HG muscles are attached was localized by palpation and transverse cuts were made approximately 4 mm lateral from the midline and 3.5–4.0 mm deep (until the fat and epithelium lining up the outer surface of the pharyngeal airway was exposed) to severe the tendons connecting the GH and HG muscles to the H apparatus. Then, a thin layer of surgical wax was inserted in the space opened up by the cut to prevent the muscles from re-connecting and the overlying mylohyoid muscle was sutured to close the cavity and keep the wax barrier in place. An additional five control rats had the connection of the GH/HG muscles to the H apparatus left intact.

For recording of nuchal EMG, two 64-stranded, teflon-coated, stainless steel wires (#AS636; Cooner Wire) were sutured to dorsal neck muscles. To record activity of the sternal diaphragm, two wires (#AS636) were passed through the sternal diaphragm on both sides of the midline and were then anchored to the cartilaginous process of the sternum. For monitoring the cortical EEG, two screws were attached to the parietal bone, 3 mm posterior and 2 mm to the right and left of bregma, and an additional screw was attached to the frontal bone to serve as electrical reference. The wires implanted into the tongue and sternal diaphragm were tunneled subcutaneously toward the head and, together with the nuchal wires and those attached to the EEG screws, were connected to a mini-socket (220-9 pin ABS plug, Ginder Scientific, Ottawa, ON, Canada) that was subsequently attached to the skull with acrylic dental cement. All skin openings were tightly sutured, and the animal was given antibiotic (Gentamicin, 5 mg/kg, i.m.), stimulant (Yohimbine, 5.0 mg/kg, i.m.), and analgesic (Metacam, 2 mg/kg, i.m.).

### Habituation and recording procedures

On day 5 after instrumentation, each animal was habituated to the recording conditions by placing it inside its home cage in a ventilated, dimly illuminated, and sound-attenuated recording chamber for 2–4 h. Then, on day 6 and thereafter, the animal was briefly immobilized to attach the recording cable to the head socket and placed in its home cage for recording. During the recording on day 6, optimal gains for all signals were established and then the first record designated for analysis was collected. The gains were set individually for each signal to obtain maximal amplification without saturation of the amplifiers at times of maximal activity and were kept constant during all subsequent recording sessions. The EEG and nuchal and diaphragmatic EMGs were recorded differentially, whereas the signals from each wire implanted into the tongue were recorded in a monopolar configuration against a reference point on the skull because this allowed for subsequent unequivocal identification of each recording site within the tongue.

Cortical EEG and lingual, nuchal, and diaphragmatic EMGs were amplified (Model 8-10B amplifiers, Grass, Warwick, RI, USA) with a bandwidth of 0.3–100 Hz for the EEG and 30–1000 Hz for the EMGs. All signals were digitally stored using sampling rates of 100 and 1000 Hz, respectively (Power-1401 A/D converter and Spike-2 v.7 data acquisition system; Cambridge Electronic Design, Inc., Cambridge, England). During data collection, the animal was left undisturbed with free access to food and water. With the exception of the recording on day 6, all data used in this report were obtained from the middle 2 h of a 4.0 h-long recording session conducted between noon and 4 p.m. on days 8, 11, 14, and 22 after instrumentation. Some of the recordings on day 6 that were used for analysis were 1.5 h-long because relatively longer time was needed to establish satisfactory gains of the amplifiers. The first hour of all recordings was excluded from analysis to allow the animal to settle down after it was connected to the recording system.

### Signal processing, scoring of sleep–wake states, and data analysis

Prior to analysis, EEG and EMG signals were digitally filtered using a band-pass filter set at 1.25–40 Hz for the EEG, and high-pass filtered at 125 Hz for the EMGs to eliminate any EEG contamination from monopolar recordings of lingual EMG and minimize the electrocardiogram present in diaphragmatic EMG.

Behavioral states were scored in successive 10 s epochs as wakefulness, SWS, or REMS based on the appearance of the cortical, nuchal, and diaphragmatic signals and the simultaneous display of the power spectrum of the cortical EEG using sleep-scoring software (Somnologica; Medcare, Buffalo, NY, USA). SWS was recognized based on the presence of a high-amplitude/low-frequency EEG, tonic nuchal EMG at a low level, and regular respiratory rate. Recognition of REMS was based on transition of the cortical EEG to a low-amplitude and high frequency, further reduction of nuchal EMG (atonia) with occasional twitches, and irregular diaphragmatic activity. To reduce overestimation of EMG levels during sleep introduced by large bursts of activity associated with awakenings, the epochs in which awakenings occurred were scored as SWS or REMS only when these states occupied at least 75% of the duration of the epoch; all other epochs were scored according to the state that occupied more than 50% of the epoch duration. After scoring, root mean square (RMS) values of the EMG signals were calculated within successive scoring epochs and exported to a spreadsheet together with concurrently calculated cortical EEG powers in selected bands (delta-2: 1.25–2.0 Hz; delta-1: 2.0–4.5 Hz; theta: 5.0–8.0 Hz; beta-2: 20–25 Hz). Two procedures were then used to verify the accuracy of scoring. First, a hypnogram was plotted for the entire recording session together with the plots of EEG delta-1 and theta powers, the beta-2/delta-2 power ratio, and the RMS values for nuchal and lingual EMGs. Second, scatter plots of RMS values for nuchal and lingual EMGs vs. the beta-2/delta-2 band EEG power ratio for all scoring epochs were constructed to verify that they exhibit distinct clustering by behavioral states (28, 29). Any data points suggesting scoring inconsistencies were re-inspected and, if appropriate, scoring was refined accordingly. No data segments were removed from the continuous records designated for analysis.

For EMG quantification, the level of signal corresponding to electrical noise only was defined as the lowest RMS value among all analyzed 10 s epochs in each recording session and this value was subtracted from RMS values for all epochs (28). All data sets were then sorted by behavioral state and the mean levels of lingual and nuchal EMGs were calculated separately for wakefulness, SWS, and REMS. To track any systematic changes in EMG activity across successive recording days, we analyzed absolute RMS levels measured in microvolt second. For additional comparison of the levels of EMG activities across the animals and recording days, EMG levels during SWS and REMS were also normalized within each recording session by their mean levels during wakefulness.

Quantitative analysis of EMG signals is not conducted often and, when this is done, different labs use different methodologies that typically are not geared toward analysis of EMG data across multiple days in multiple subjects. To date, it has not been determined whether EMG signals recorded with chronically implanted wire electrodes maintain stable levels across multiple days, which is an important requirement if one intends to measure experimentally induced changes in EMG signals over time and across subjects. Therefore, prior to analysis of lingual EMG in rats with surgically altered upper airway, we tested whether EMG signals exhibited satisfactory stability when recorded on days 6 through 22 after instrumentation using data from five rats with intact upper airway.

### Analysis of respiratory modulation of lingual EMG and respiratory instabilities

In consideration of the possibility that respiratory modulation of lingual EMG or the incidence of respiratory instabilities might be altered by our surgical intervention or depend on the time after instrumentation, two recording sessions (days 6 and 22 after instrumentation) were examined for the presence of respiratory modulation of lingual EMG and the occurrence of various instabilities of diaphragmatic activity. Respiratory modulation was analyzed as described previously (30). Lingual and diaphragmatic EMGs were rectified and integrated (time constants 50 and 100 ms, respectively), superimposed, and successive 30 s intervals of each record were sequentially examined. Lingual EMG was deemed respiratory-modulated when the integrated diaphragmatic and lingual EMGs exhibited the same rhythm and a fixed phase relationship over at least five successive respiratory cycles.

To detect transient respiratory instabilities, we focused on expiratory pauses in integrated diaphragmatic activity lasting at least 2 s (31–33). Once identified, the events were further classified as central (when not preceded by any distinct perturbations of diaphragmatic activity within at least 5 s), post-sigh (when immediately preceded by a single inspiratory burst in the diaphragm that was at least twice larger than the adjacent bursts), or other apneic events preceded within <5 s by complex breath-to-breath variations of diaphragmatic activity. We also recorded the occurrence of sighs that were not followed by apneas, but the sigh-like events associated with major body movements (large bursts of nuchal EMG) that often occur at the time of arousal from sleep were excluded from analysis.

### Statistical analysis

For normally distributed data sets, repeated measures analysis of variance (RM ANOVA), followed by Student’s *t*-test with Holm–Sidak correction for multiple comparisons was used to compare EMG levels among behavioral states, recording days, and treatments. For analysis of respiratory modulation of lingual EMG and transient breathing instabilities, Student’s *t*-test or Wilcoxon signed rank test was used. The variability of the means is characterized by the SE throughout the report. Differences were considered significant at *p* < 0.05.

### Histological verification of lingual recording sites and effectiveness of separation of the GH/HG muscles from the H apparatus

At the end of the study, the animals were deeply anesthetized with isoflurane (5%) followed by pentobarbital (Nembutal; 100 mg/kg, i.p.) and decapitated. The head was fixed in 10% formalin and the tongue was then manually sliced *in situ* in sagittal plane and the locations of the tips of recording wires were identified. These sites were then plotted onto a standard, sagittal cross-section of the rat tongue. The entire upper airway segment extending from the level below the H apparatus upward to the pharynx, including all muscles surrounding the upper airway, trachea, and esophagus, was then extracted and serially cut on a cryostat (CM 1850, Leica Microsystems, Nussloch, Germany) into 35 μm parasagittal sections. Sections were stained with neutral red and inspected to verify that the wax barrier effectively prevented GH and HG muscles from re-connecting to the H apparatus and compare upper airway anatomy in the intact and experimental rats.

## Results

The rats with GH/HG muscles disconnected from the H apparatus did not exhibit any overt problems with breathing when under anesthesia during instrumentation or thereafter, nor did they appear to have any problems with eating or drinking. Between the day of instrumentation and the last recording day (day 22), the rats with GH/HG muscles disconnected from H apparatus gained 112 ± 12 g (SE) in body weight, and the rats with the GH/HG connection intact gained 84.0 ± 8.9 g (not significantly different). The somewhat larger body weight gain in experimental rats could be related to their lower starting body weight at the time of instrumentation 316 ± 25 vs. 371 ± 16 g for the rats with intact upper airway (*p* = 0.09) and the fact that the rate of body weight gain decreases with age.

**Figure 1:**
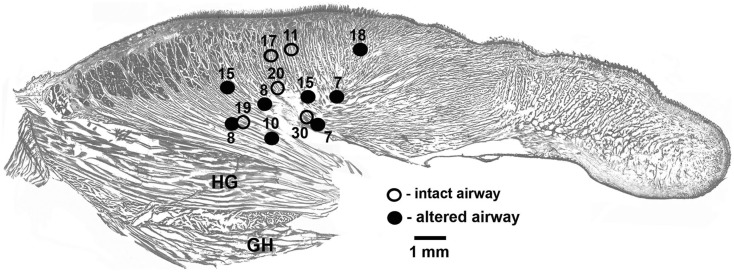
**Location of the recording sites in the tongue in the rats of this study**. The sites were localized post-mortem and superimposed on a standard sagittal cross-section of the rat tongue. Lingual EMG signals recorded from eight sites in five rats with GH/HG muscles disconnected from H apparatus (filled circles) and from five recording sites in five rats with intact upper airway (open circles) were analyzed. When signals from two sites in the same animal were available, the mean EMG levels recorded from the two sites were averaged to ensure that each animal contributed evenly to the group mean data. The numbers identify different animals.

In each of the five rats with intact upper airway, one lingual recording site was localized in the proximal pharyngeal or basal part of the tongue, as intended. In three of the five rats with altered upper airway, two recording sites were localized in the proximal part of the tongue near its base and one such site was found in each of the remaining two rats. To maintain a balanced design, with each rat contributing one data set characterizing lingual EMG, lingual activity recorded from the two rats that had both leads satisfactorily placed in the pharyngeal region of the tongue was averaged between the two leads and treated as one data set. Figure 1 shows the location of all lingual recording sites in the control rats (five sites in five animals) and the rats with GH/HG muscles disconnected from H apparatus (eight sites in five animals).

### Stability of lingual and nuchal EMGs on successive recording days after instrumentation in rats with intact upper airway

**Figure 2:**
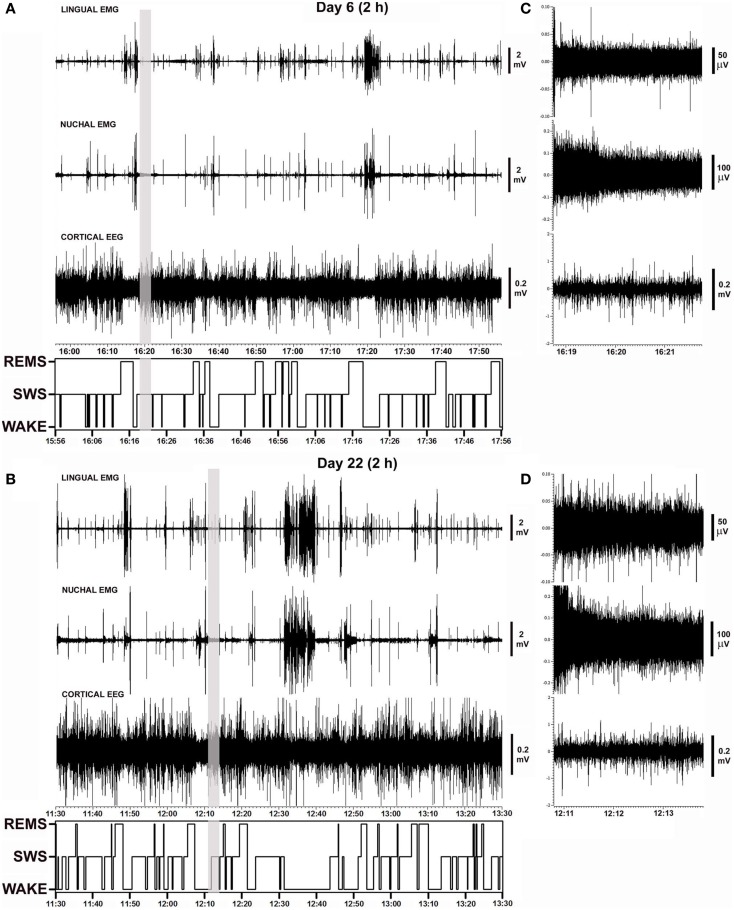
**Examples of polygraphic records collected on days 6 and 22 after instrumentation from a rat with intact upper airway (rat 17 in Figure 1)**. **(A,B)** Continuous records of lingual and nuchal EMGs and cortical EEG, and the corresponding hypnograms spanning the entire analyzed recording periods (2 h). Periods with low levels of lingual and nuchal EMG during quiet wakefulness and sleep are interrupted by intense bursts of activity associated with active wakefulness. **(C,D)** Nine minutes-long segments of the records marked by the vertical gray lines in **(A,B)** shown using expanded time and amplitude scales. The segments are taken from SWS periods when lingual muscles are atonic or nearly atonic. The records demonstrate a close similarity of activity patterns, sleep–wake patterns, and noise levels recorded from the same animal 16 days apart.

Figure 2 shows polygraphic records of lingual and nuchal EMGs and cortical EEG with the corresponding hypnograms that span 2 h obtained from the same animal on days 6 and 22 after instrumentation. The records demonstrate a close similarity of the amplitudes and patterns of all signals recorded from the same animal 16 days apart. The sleep–wake pattern (hypnogram) on day 6 is also similar to that on day 22. The gray vertical lines in Figures 2A,B mark the segments of record shown in Figures 2C,D using expanded gains and time scales. The expanded segments show EEG and EMG signals during SWS when lingual EMG is very low or absent, nuchal EMG is steady at low tonic level, and cortical EEG is of high amplitude. As such, these records demonstrate that the electrical noise level and the signal levels were of comparable amplitudes on both recording days.

**Figure 3:**
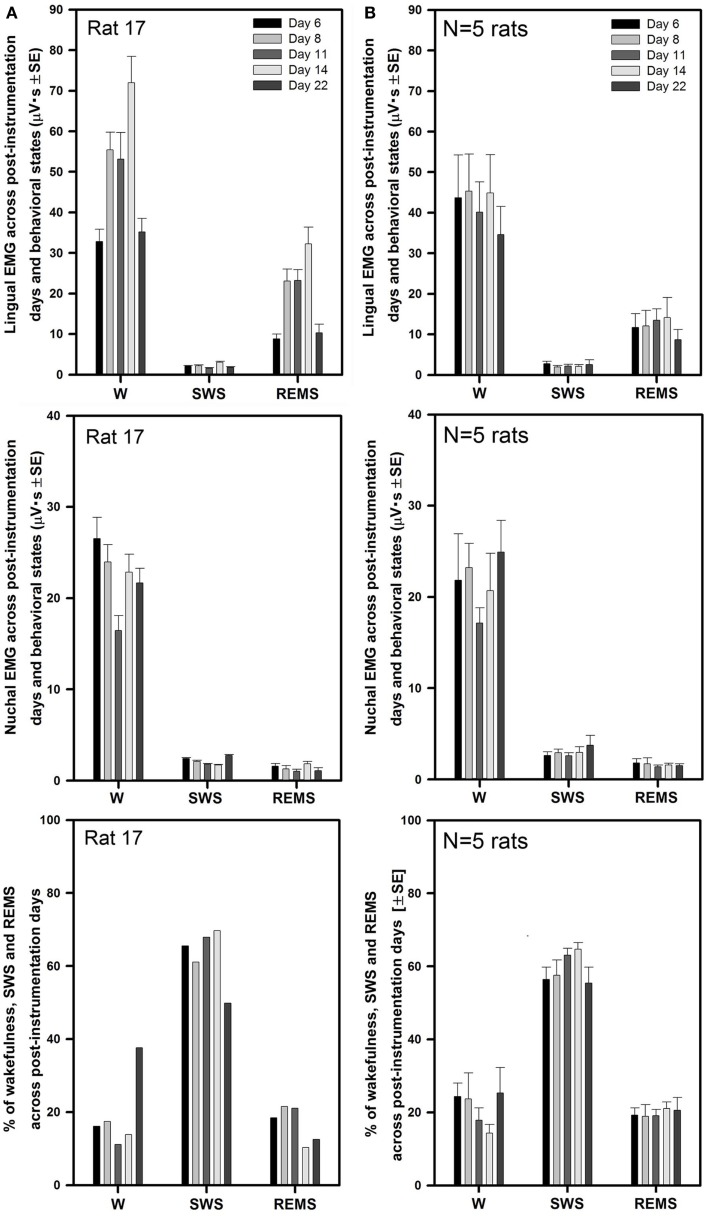
**Stable levels of lingual and nuchal EMGs across successive recording days in rats with intact upper airway**. **(A)** Lingual and nuchal EMG levels quantified separately during different sleep–wake states, and the percentage amounts of wakefulness, SWS, and REMS in one rat across all five recording sessions conducted on days 6, 8, 11, 14, and 22 after instrumentation (rat 17 in Figure 1). **(B)** Average levels of lingual and nuchal EMG, and the mean percentage amounts of wakefulness, SWS, and REMS in five rats with intact upper airway studied on the same 5 days after instrumentation. There was no systematic, time-dependent trend in the levels of lingual or nuchal EMGs, or the amount of sleep–wake states across the 16 days separating the first and last recording session.

Figure 3A illustrates the mean lingual and nuchal EMG levels quantified separately during different sleep–wake states and the percentage amounts of wakefulness, SWS, and REMS in one rat across all five recording sessions conducted on days 6 through 22 after instrumentation (same animal as in Figure 2). The mean levels of lingual EMG during wakefulness varied among the recording sessions by not more than 45% of the mean across all days and there was no systematic, time-dependent trend (Figure 3A, top panel). It is of note that the mean lingual EMG was at its lowest level consistently during SWS, whereas during REMS it increased as a result of frequent large twitches that characterize lingual activity during this state (28, 29, 34). Similarly, nuchal EMG measured during wakefulness varied from day to day by <28% of the mean across all days (Figure 3A, middle panel). The percentage amounts of different behavioral states varied only slightly among the recording days except a relatively larger amount of wakefulness on day 22 (11–38% for wakefulness, 50–70% for SWS, and 13–22% for REMS; Figure 3A, bottom panel).

As illustrated for one animal in Figure 3A, the average lingual and nuchal EMGs calculated across all five rats with intact upper airway shown in Figure 3B also were quite stable across all recording days. For all five control rats, the mean lingual EMG levels measured in microvolt second varied across all five recording sessions from 35 ± 7 on day 22 to 45 ± 9 on day 8 during wakefulness, from 1.9 ± 0.3 on day 6 to 2.7 ± 0.6 on day 8 for SWS, and from 8.7 ± 2.5 on day 22 to 12 ± 3.9 on day 6 for REMS (top panel). The levels of lingual EMG measured in absolute units within each behavioral state did not differ among the recording days (one-way RM ANOVA and Friedman RM ANOVA on ranks), and no systematic trend was evident. The mean levels of lingual EMG across all five recording days for all five rats were (in microvolt second): 42.0 ± 3.8 for wakefulness, 2.3 ± 0.3 for SWS, and 12.2 ± 1.6 for REMS.

The mean nuchal EMG in the five control rats measured in microvolt second varied across all five recording sessions from 17 ± 2 on day 11 to 25 ± 4 on day 22 during wakefulness, from 2.6 ± 0.3 on day 11 to 3.7 ± 1.1 on day 22 for SWS, and from 1.4 ± 0.2 on day 11 to 1.8 ± 0.4 on day 6 for REMS (Figure 3B, middle panel). One-way RM ANOVA applied separately to data during wake, SWS, and REMS did not reveal any significant differences in nuchal EMG levels among the recording days. The mean levels of nuchal EMG across all five recording days for all five rats were (in microvolt second): 21.4 ± 1.6 for wakefulness, 2.9 ± 0.25 for SWS, and 1.6 ± 0.2 for REMS.

The average percentage amounts of different behavioral states did not differ across the recording days (one-way RM ANOVA). The range of the average percentages of recording time spent in different behavioral states varied from 14 ± 2% on day 14 to 25 ± 7% on day 22 for wakefulness, from 55 ± 4% on day 22 to 65 ± 2% on day 14 for SWS, and from 19 ± 3% on day 8 to 21 ± 2% on day 14 for REMS (Figure 3B, bottom panel).

### Lingual and nuchal EMG levels across behavioral states and time after instrumentation in rats with GH and HG muscles disconnected from the H apparatus

**Figure 4:**
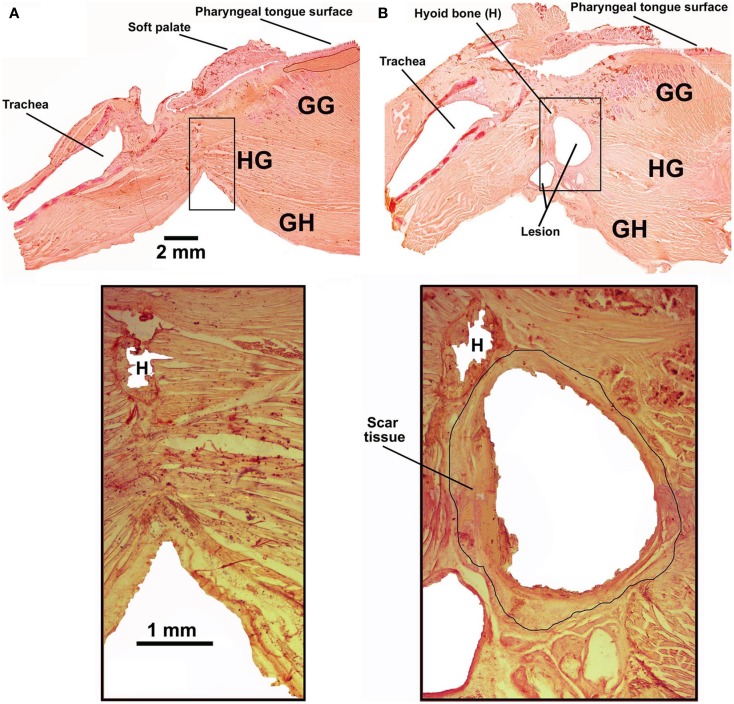
**Examples of parasagittal cross-sections through the upper airway in an intact rat [(A); rat 11 in Figure 1] and a rat with the GH/HG muscles disconnected from the H apparatus [(B); rat 8 in Figure 1]**. The bottom panels show enlarged images of the region where the cut was made and wax barrier inserted to prevent re-connection in the rats with compromised upper airway. In all rats with altered upper airway, the wax barrier remained in place and GH/HG muscles ended with a healed scar [black circle around the lesion in the enlarged image in **(B)**], with no evidence of re-connection to their original insertion point.

Five rats had the tendon connecting the GH/HG muscles to H apparatus surgically severed at the time of instrumentation and a wax barrier inserted to prevent re-connection. Figure 4 illustrates parasagittal sections through the upper airway of a control rat (Figure 4A, rat 11 of Figure 1) and a surgically altered rat (Figure 4B, rat 8 of Figure 1). In all rats with altered upper airway, the wax barrier remained in place, and GH/HG muscles ended with a healed scar with no evidence of re-connection to their original insertion site.

**Figure 5:**
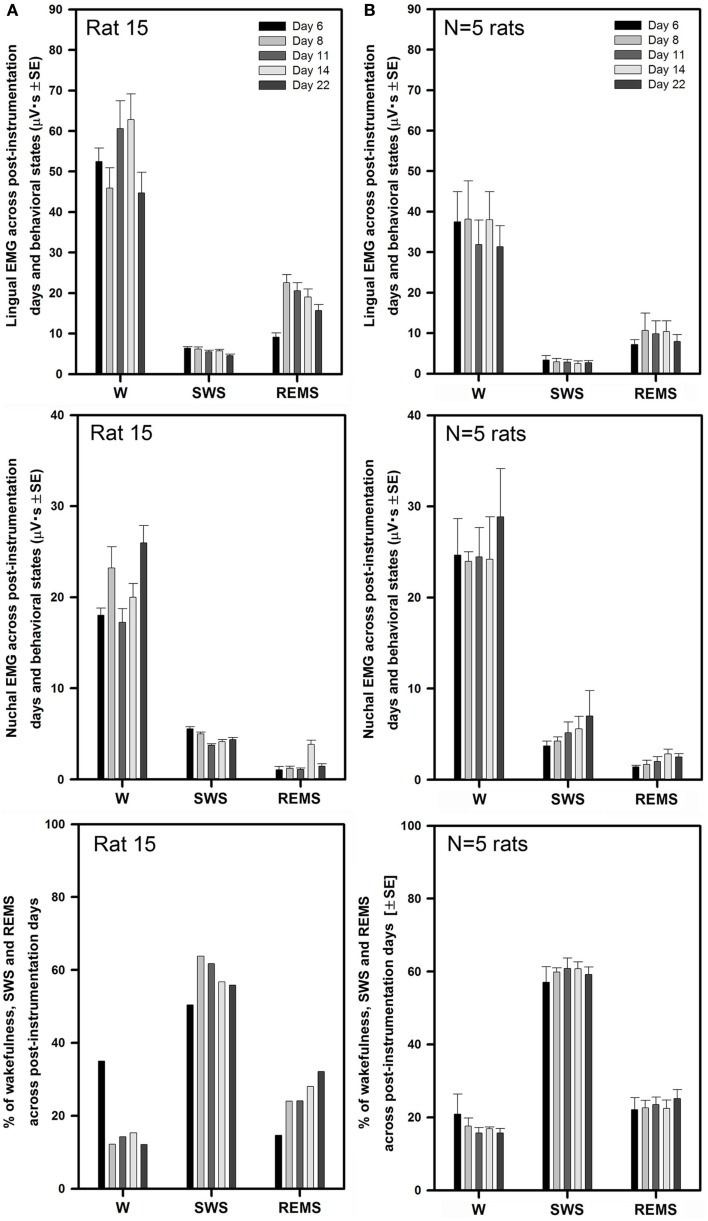
**Stable levels of lingual and nuchal EMGs across successive recording days in rats with GH/HG muscles disconnected from the H apparatus**. **(A)** Lingual and nuchal EMG levels quantified separately during different sleep–wake states, and the percentage amounts of wakefulness, SWS, and REMS in one rat across all five recording sessions conducted on days 6, 8, 11, 14, and 22 after instrumentation (rat 15 in Figure 1). **(B)** Average levels of lingual and nuchal EMG, and the mean percentage amounts of wakefulness, SWS, and REMS in the five rats with compromised upper airway studied on the same 5 days after instrumentation. Within this data set, the level of nuchal EMG during REMS was higher on day 14 when compared to day 6 (*p* = 0.04); other than that, no significant effects of the recording day were detected.

Similarly to the rats with intact upper airway (Figures 2 and 3), lingual and nuchal EMGs of the rats with altered upper airway were stable across all recording days. Figure 5A shows the mean lingual and nuchal EMG levels quantified separately during different sleep–wake states and the percentage amounts of wakefulness, SWS, and REMS across all five recording sessions conducted on days 6 through 22 after instrumentation in one rat with altered upper airway (rat 15 of Figure 1). Lingual EMG during wakefulness varied from day to day by not more than 18% of the mean across all days (top panel), and nuchal EMG by not more than 24% (middle panel). In this rat, the percentage amounts of different behavioral states varied only slightly except the relatively larger amount of wake on day 6 and an increasing trend for REMS across all days. The percentage amounts of different vigilance states on different recording days were: 12–35% for wakefulness, 50–64% for SWS, and 15–32% for REMS (bottom panel).

For all five rats with altered upper airway, the mean lingual EMG measured in microvolt second varied among the recording sessions from 31 ± 5 on day 22 to 38 ± 9 on day 8 for wakefulness, from 2.5 ± 0.7 on day 14 to 3.4 ± 1.1 on day 6 for SWS, and from 7.2 ± 1.2 on day 6 to 11 ± 4.2 on day 8 for REMS (Figure 5B, top panel). When compared within different behavioral states, lingual EMG levels did not differ among the recording days (one-way RM ANOVA and Friedman RM ANOVA on ranks). The mean levels of lingual EMG across all five recording days for all five rats were (in microvolt second): 35.4 ± 3.0 during wakefulness, 2.9 ± 0.3 during SWS, and 9.2 ± 1.2 during REMS.

The mean nuchal EMG measured in microvolt second varied among the recording sessions from 24 ± 1 on day 8 to 29 ± 5 on day 22 for wakefulness, from 3.7 ± 0.5 on day 6 to 6.9 ± 2.8 on day 22 for SWS, and from 1.4 ± 0.2 on day 6 to 2.8 ± 0.5 on day 14 for REMS (Figure 5B, middle panel). There was a trend for a gradual increase of nuchal EMG during SWS between days 6 and 22 after instrumentation. One-way RM ANOVA revealed significant effect of recording day on the level of nuchal EMG during REMS (*F*_4,4,16_ = 3.88, *p* = 0.02), which was due to a particularly high level of nuchal EMG on day 14 when compared to day 6 (*t* = 3.411, *p* = 0.04). No such trends occurred in the rats with intact upper airway (Figure 3B, middle panel). The mean levels of nuchal EMG across all five recording days for all five rats were (in microvolt second): 25.2 ± 1.7 for wakefulness, 5.1 ± 0.7 for SWS, and 2.1 ± 0.2 for REMS.

The average percentage amounts of different behavioral states did not differ among the recording days in the rats with altered upper airway (one-way RM ANOVA). The mean percentages of recording time spent in different behavioral states varied from 16 ± 1% on day 11 to 21 ± 5% on day 6 for wakefulness, from 57 ± 4% on day 6 to 61 ± 3% on day 11 for SWS, and from 22 ± 3% on day 6 to 25 ± 2% on day 22 for REMS (Figure 5B, bottom panel).

### Comparison of lingual and nuchal EMG levels across behavioral states and time after instrumentation in rats with intact and altered upper airway

**Figure 6:**
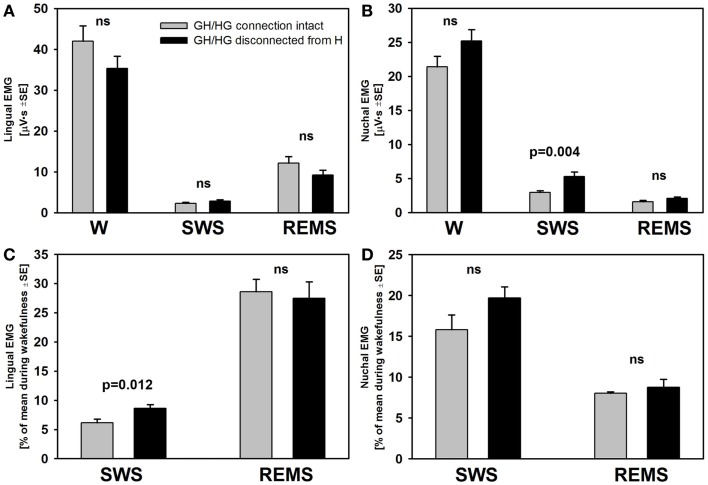
**The mean lingual and nuchal EMG levels during different sleep–wake states in control rats and rats with GH/HG muscles disconnected from H apparatus**. When quantified in absolute units, both lingual **(A)** and nuchal **(B)** EMGs had similar characteristic patterns of mean levels of activity across wakefulness (W), SWS, and REMS in the control and experimental rats, with only nuchal EMG measured during SWS being significantly higher in the experimental than in the control rats. The rats subjected to surgical intervention tended to have reduced lingual EMG and elevated nuchal EMG during W, suggesting a protective reaction to the injury applied to the ventral neck region. When lingual and nuchal EMG levels during sleep were quantified relative to their levels during wakefulness **(C,D)**, lingual EMG during SWS was significantly higher in the rats with altered upper airway when compared to control rats, whereas there was only an insignificant trend in this direction for nuchal EMG. See text for alternative interpretations of this finding. Bars show average data derived from 24 recording sessions with 5 control rats and 25 recording sessions with 5 experimental rats. ns – not significant difference between the control and experimental rats.

There was no difference across the recording sessions in the amounts of wake, SWS, or REMS between the intact and experimental animals. Two-way RM ANOVA applied to lingual EMG levels measured in absolute units during wakefulness, SWS, and REMS across all rats and all recording days did not reveal any significant effects of surgical intervention. Nevertheless, it is of note that the rats with GH/HG muscles disconnected from the H apparatus tended to have relatively lower levels of lingual EMG and relatively higher levels of nuchal EMG during wakefulness than the control animals. Nuchal EMG also was significantly higher in the experimental rats during SWS (5.1 ± 0.7 vs. 2.9 ± 0.25 μVs, *p* = 0.004, df = 47, *t*-test), whereas this difference was not significant for lingual EMG (Figures 6A,B). These trends and differences in absolute levels of lingual and nuchal EMGs between the two groups may have reflected different reactions of these muscles to the surgical intervention (see Discussion).

The differences in absolute EMG levels may have also contributed to the differences between the two groups detected when lingual and nuchal EMG levels during SWS and REMS were measured relative to their levels during wakefulness (Figures 6C,D). When such a relative calculation was used and data from all five recording sessions from all rats in each group were averaged, the mean relative level of lingual EMG was significantly higher during SWS in the rats with altered upper airway when compared to the control animals (8.6 ± 0.7 vs. 6.1 ± 0.7%, *p* = 0.012, df = 47, *t*-test). The relative levels of nuchal EMG during SWS also tended to be higher in the experimental rats 19.7 ± 1.4 vs. 15.8 ± 1.8%, but the difference was not statistically significant (*p* = 0.09, df = 47, *t*-test). When the relative levels of lingual and nuchal EMGs during SWS were compared by first calculating the means across all recording sessions within each rat and then comparing the means between the groups of five control and five experimental rats, there was only a trend for a higher relative lingual EMG level in experimental rats (8.6 ± 1.4 vs. 6.2 ± 0.9%, *p* = 0.08, df = 8, *t*-test), and no evidence of such an effect for nuchal EMG (19.7 ± 1.3 vs. 16.2 ± 3.8%, *p* = 0.4, df = 8, *t*-test). The corresponding analysis of the effect of surgical intervention on the levels of lingual and nuchal EMGs during REMS did not reveal any significant effects (Figures 6C,D).

### Inspiratory modulation of lingual EMG in rats with intact and altered upper airway

**Figure 7:**
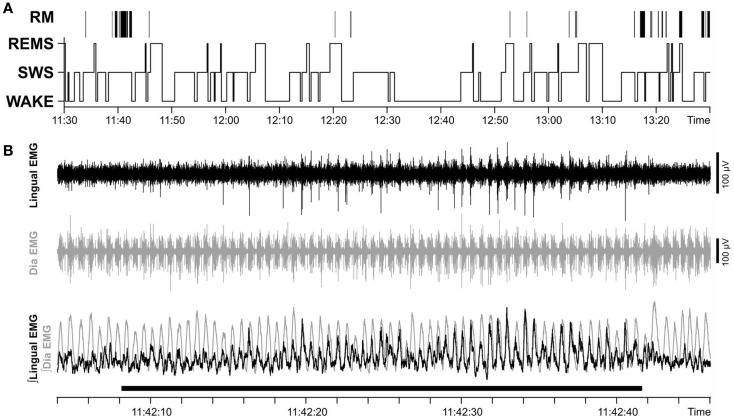
**Respiratory modulation of lingual EMG was rare and occurred mainly during SWS**. **(A)** Distribution of inspiratory-modulated segments of lingual EMG in relation to sleep–wake states in a 2 h-long recording sessions obtained on day 22 after instrumentation (rat 17 of Figure 1). Positions of the segments with respiratory modulation (RM) of lingual EMG are marked by vertical lines above the hypnogram. The RM segments occurred mainly during SWS, lasted 2.4–62.2 s, and collectively occupied 2.4% of the entire recording session. **(B)** Example of a record from the session illustrated in **(A)** during which inspiratory modulation appeared and then faded away during a period of SWS. The record is 44 s-long and shows the raw and integrated lingual and diaphragmatic (Dia) EMGs. Superposition of the integrated EMGs from both muscles (bottom trace) reveals that lingual EMG is inspiratory-modulated with a stable phase relationship relative to diaphragmatic activity during the 32 s-long period marked by the horizontal bar at the bottom.

In chronically instrumented, intact rats, respiratory modulation of lingual EMG is usually of the inspiratory type but occurs relatively rarely (30). We hypothesized that our surgical intervention could increase the occurrence of inspiratory modulation of lingual EMG. However, our analysis revealed that the incidence of inspiratory modulation was similar in both groups of animals. Figure 7A shows the distribution of inspiratory-modulated segments of lingual EMG during 2 h-long recording sessions obtained from rat 17 of Figure 1 on day 22 after instrumentation, and Figure 7B illustrates an example of a segment of record from the session illustrated in Figure 7A during which inspiratory modulation appeared and then faded away during a period of SWS.

In the control rats, the cumulative duration of inspiratory-modulated segments per hour of recording varied from 1.5 to 697 s [mean 140 ± 96 s; median 7.0 s, inter-quartile range (IQR) 2.8–182.6 s], and the frequency of segments was 5.6 ± 2.5 h^−1^ (median 2.5 h^−1^, IQR 0.88–11.38 h^−1^). In the experimental rats, the cumulative duration per recording hour varied from 0 s (one animal did not have a single segment of inspiratory-modulated lingual EMG in either recording session) to 136 s (mean 25 ± 16 s; median 8.3 s, IQR 1.3–23.3 s), and the frequency of segments was 2.3 ± 1.0 h^−1^ (median 1.8 h^−1^, IQR 0.25–3.0 h^−1^). Neither the cumulative duration nor the frequency of occurrence of inspiratory-modulated segments differed between the control and experimental rats (*p* = 0.61 and 0.28, respectively).

The total duration of all inspiratory-modulated segments was 41 min and 21 s, corresponding to 2.4% of the entire duration of the examined records (29 h). This time comprised 110 separate segments with inspiratory-modulated lingual EMG of which nearly all (100 segments, or 90.9%) occurred during SWS (Figure 7). Typically, inspiratory modulation started with a delay after the onset of SWS and ended at or before the end of the same SWS episode.

### Respiratory instabilities in rats with intact and compromised upper airway

**Figure 8:**
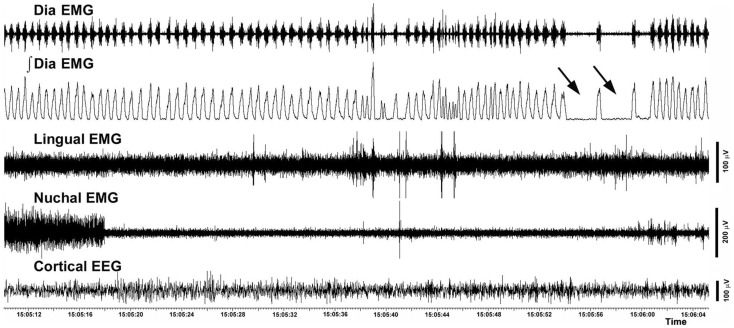
**Example of two successive central apneic events during REMS**. The traces show a 56 s-long record of the raw and integrated diaphragmatic EMG, raw lingual and nuchal EMGs and EEG. Arrows point to two apneas longer than 2 s. The record was collected on day 22 after instrumentation from a rat with altered upper airway (rat 10 in Figure 1).

The incidence of different categories of respiratory instabilities was compared among the recording days and experimental and control groups during wakefulness, SWS, and REMS. There was no effect of the recording day, and we found no difference between the rats with intact and compromised airway. Figure 8 shows an example of two successive apneas lasting longer than 2 s that occurred during a REMS period.

The total number of all distinct events identified on both recording days in both groups of animals during a total of 36 h of analyzed recordings was 338. Of those, 230 were apneas preceded by complex diaphragmatic breath-to-breath variability; they were most common during wakefulness (203; mean 23 ± 2.7 per animal; range 12–36) and much less frequent during SWS (20; mean 2.2 ± 0.8; range 0–7) or REMS (7; mean 0.8 ± 0.3; range 0–3). Similarly, most post-sigh apneas occurred during wakefulness (34; mean 3.8 ± 1.6; range 0–13), and only a few during SWS (9; mean 1.0 ± 0.5; range 0–4) or REMS (4; mean 0.4 ± 0.3; range 0–3). The mean frequency of all types of apneas during REMS was 6.3 ± 2.1 h^−1^, and the mean frequency of apneas during sleep (SWS + REMS) was 2.0 ± 0.5 h^−1^. The latter was similar to an earlier report (31). The total number of sighs not followed by apneic events was 61. Of those, 44 occurred during wakefulness, 13 during SWS, 3 during REM, and 1 at awakening from SWS. The total number of apneic episodes not preceded by sigh or other diaphragmatic perturbations was 61, with the highest incidence of such events during REMS (46; mean 5.1 ± 1.6; range 0–13), and much lower incidence during either wakefulness (9; mean 1.0 ± 0.4; range 0–4) or SWS (6, mean 0.8 ± 0.3; range 0–2). Among the 46 apneic episodes observed during REMS, 30 (mean 6.0 ± 2.3; range 2–13) occurred in rats with compromised airway and 16 in intact rats (mean 4.0 ± 2.4; range 0–11) but the trend toward a higher incidence of apneic episodes in rats with compromised airway was not statistically significant.

## Discussion

We found that surgical disconnection of the GH and HG muscles from the hyoid apparatus results in an increase of lingual muscle activity during SWS when quantified relative to the mean level of activity during wakefulness. The increase was relatively modest and not associated with increased respiratory modulation of lingual muscle activity or increased occurrence of instabilities of respiratory rhythm, indicating that the surgical intervention tested did not significantly compromise upper airway patency. We also found that the incidence of central apneic episodes was highest during REMS, whereas other types of apneas (post-sigh and those following complex variations of diaphragmatic activity), as well as sighs not followed by apnea were mostly frequent during wakefulness. In the course of this study, we also determined that recordings from upper airway and nuchal muscles conducted repeatedly over a period from 6 to 22 days after instrumentation using chronically implanted wire electrodes yield consistent day-to-day measures of EMG activity levels, thus the approach can be used for longitudinal, quantitative studies combined with interventions that may elicit systematic changes in muscle activity.

### Lingual muscle activity across sleep–wake states in rats with intact and compromised upper airway

Most animals used as models in OSA-related studies do not have collapsible upper airway and do not experience sleep-related upper airway obstructions. Since rodents (rats and mice) are commonly used to study the neural control of sleep and breathing, there is a great need to develop a satisfactory model of OSA in rodent species. It has been shown that obese rats and mice have narrower and more collapsible upper airway than their lean counterparts (18, 19, 35), and one study suggested that rats sleeping in a tightly curled position may experience flow limitation because this body position was associated with more pronounced appearance of inspiratory modulation of lingual EMG (36). However, to date, there is no evidence that spontaneous, sleep-related obstructive events occur in rodents. Furthermore, recordings of lingual EMG from obese Zucker rats did not reveal an increased level of upper airway muscle tone when compared to lean rats, as would be expected should the increased fat accumulation lead to significant flow limitations that would necessitate stronger muscle contraction to maintain the patency of the upper airway (20).

The GH and HG muscles due to their anatomical location provide a passive mechanical support to the upper airway in the pharyngeal region and act synergistically with the GG when the upper airway needs active support. Accordingly, we reasoned that disconnecting GH and HG muscles from their attachment to the H apparatus would make the upper airway of a rat more collapsible and potentially impose additional demand on the force exerted by the GG muscle. We determined that, when quantified across all recording days relative to the mean activity level during wakefulness, lingual EMG was modestly but significantly increased during SWS in surgically altered rats when compared to the control group. One possible interpretation of this result is that a weakened support of the upper airway caused by our surgical intervention elicited a compensatory increase of activity in other synergistic muscles that were left intact. However, the increase of lingual EMG that we detected during SWS in the rats with compromised upper airway was significant only when the level of activity was expressed relative to the level of activity during wakefulness. Furthermore, although the augmentation detected by this metrics represented a 38% increase when compared to the control rats, its magnitude in absolute units was small. By comparison, GG activity in severe OSA patients measured during wakefulness is about four times higher than in age- and body weight-matched control subjects (5), and sternohyoid muscle activity in English bulldogs measured during SWS is nearly three times higher than in control dogs (37). Interestingly, upper airway muscle tone was also acutely reduced in OSA patients by graded mandibular advancement when measured during wakefulness (38). Thus, there are both central and reflex mechanisms that mediate the compensatory changes in the level of upper airway muscle tone that can be elicited by experimental manipulations such as recurrent hypoxia or flow limitation, and their dynamic range seems to be larger that the effects observed in our present study.

Our assessment of the incidence of inspiratory modulation of lingual EMG and respiratory rhythm perturbations (apneas) also did not indicate that the intervention that we tested resulted in significant flow limitations, let alone obstructions. It is possible that the cuts that we made were insufficient to reach a threshold at which upper airway becomes vulnerable to collapse. An analogous situation has been reported in experiments with balloons inserted into the lateral walls of the upper airway in mini pigs with the goal to experimentally produce flow limitations (39). In those experiments, respiratory perturbations occurred only after the balloons were inflated above a threshold volume. In our experiments, the muscles and other tissues surrounding the upper airway that were left intact must have been sufficient to ensure the stability and patency of the upper airway despite the cut of the connection between the GH and HG muscles and the H apparatus. It is also possible that multiple muscles participated in the compensatory reaction to our surgical intervention, as it is the case in patients with anterior cruciate ligament injuries in whom multiple muscles partially contribute to the compensatory changes in muscle activation during locomotion (40). Indeed, the changes revealed by our analysis of the absolute levels of lingual and nuchal EMGs in experimental rats (Figures 6A,B) suggest that our intervention might have caused a reduced use of ventral neck muscles [as a protective reaction to injury; cf. Ref. (26)] with a concomitant increase of the use of dorsal neck muscles (nuchal EMG). This form of adjustment would be expected to occur primarily during wakefulness. Accordingly, our finding that the rats subjected to surgical intervention had increased lingual EMG during SWS when it was quantified relative to the level of activity during wakefulness could be primarily due to reduced lingual muscle activation during wakefulness in experimental rats (Figure 6A). This effect was not evident for nuchal EMG because its absolute levels were elevated during both wakefulness and SWS (significant during SWS only; Figure 6B). This interpretation would be consistent with elevated activation of dorsal neck muscles representing a protective mechanism in the face of injury inflicted onto ventral muscles in the neck region.

Thus, there are alternative explanations for our findings, but it is also of note that the rats well-tolerated what appeared to be a relatively extensive interference with the ability of GH and HG muscles to exert their functions. This encourages more extensive surgical attempts to weaken the mechanical support of the upper airway in rodents to produce a collapsible airway. Based on our present study, additional efforts are worth undertaking to find the most effective approach to generate a rodent model with upper airway patency significantly compromised in a state-dependent manner.

### Respiratory modulation of lingual activity and respiratory instabilities

Inspiratory modulation of upper airway muscle activity is stronger in subjects with clinical and subclinical OSA than in persons with fully patent upper airway (4, 7, 21, 23, 41). This is a beneficial adaptation that protects the upper airway from collapse at the time when negative intraluminar pressure exerts centripetal force on the airway walls (5, 22). In rats, respiratory modulation can be elicited by chemical stimulation of breathing, direct activation of XII motoneurons or vagotomy (2), but it occurs rarely under the normal, baseline conditions (28, 30, 36). Consistent with a more extensive analysis of respiratory modulation of lingual EMG in Sprague-Dawley and Wistar rats presented elsewhere (30), in this study we also observed that inspiratory modulation occurred mainly during SWS. Since we did not observe any increased incidence of respiratory modulation of lingual EMG in rats with altered upper airway, this further suggests that our surgical intervention did not significantly compromise upper airway patency.

Respiratory pauses have been quantified in rats as markers of instability of the central respiratory rhythm generator akin to central apneas (31–33, 42). In the present study, we found that apneic episodes that were not preceded by sighs or other diaphragmatic perturbations occurred most frequently during REMS. This confirms previous studies in rats showing the highest incidence of spontaneous apneas during REMS (32, 43, 44). This is also in accordance with clinical observations in tracheostomized human OSA patients in whom central apneas are particularly common during this stage of sleep (45). We also found that, unlike the spontaneous apneas, apneas preceded by multiple diaphragmatic perturbations, post-sigh apneas, and sighs without apnea were most common during wakefulness, less frequent during SWS, and rare during REMS. Thus, apneic events of distinct types and sighs have different patterns of their sleep–wake state-dependence. Importantly for the main purpose of this study, the rats with altered upper airway had a similar incidence of apneic events as the control rats. Thus, the detachment of the GH and HG muscles from the H apparatus did not cause breathing instabilities.

## Conclusion

A weakened support of lingual muscles produced by detaching the GH and HG muscles from the H apparatus resulted in a mild increase of lingual muscle activity during SWS when quantified relative to activity during wakefulness. The rats well-tolerated the intervention, which encourages further manipulations with rodent upper airway with the aim to obtain a rodent model with collapsible upper airway.

## Conflict of Interest Statement

The authors declare that the research was conducted in the absence of any commercial or financial relationships that could be construed as a potential conflict of interest.

## References

[1] Dempsey JA Veasey SC Morgan BJ O’Donnell CP Pathophysiology of sleep apnea. Physiol Rev (2010) 90:47–112 10.1152/physrev.00043.2008 20086074 PMC3970937

[2] Kubin L Davies RO Mechanisms of upper airway hypotonia. 2nd ed In: Pack AI, editor. Sleep Apnea: Pathogenesis, Diagnosis, and Treatment. St. Helier: Informa Healthcare (2011). p. 82–127

[3] Remmers JE DeGroot WJ Sauerland EK Anch AM Pathogenesis of upper airway occlusion during sleep. J Appl Physiol (1978) 44:931–8 670014 10.1152/jappl.1978.44.6.931

[4] Katz ES White DP Genioglossus activity during sleep in normal control subjects and children with obstructive sleep apnea. Am J Respir Crit Care Med (2004) 170:553–60 10.1164/rccm.200403-262OC 15172891

[5] Mezzanotte WS Tangel DJ White DP Waking genioglossal electromyogram in sleep apnea patients versus normal controls (a neuromuscular compensatory mechanism). J Clin Invest (1992) 89:1571–9 10.1172/JCI115751 1569196 PMC443031

[6] Okabe S Hida W Kikuchi Y Taguchi O Takishima T Shirato K Upper airway muscle activity during REM and non-REM sleep of patients with obstructive apnea. Chest (1994) 106:767–73 8082357 10.1378/chest.106.3.767

[7] Suratt PM McTier RF Wilhoit SC Upper airway muscle activation is augmented in patients with obstructive sleep apnea compared with that in normal subjects. Am Rev Respir Dis (1988) 137:889–94 10.1164/ajrccm/137.4.889 3354997

[8] Yaggi HK Strohl KP Adult obstructive sleep apnea/hypopnea syndrome: definitions, risk factors, and pathogenesis. Clin Chest Med (2010) 31:179–86 10.1016/j.ccm.2010.02.011 20488280

[9] Young T Palta M Dempsey J Skatrud J Weber S Badr S The occurrence of sleep-disordered breathing among middle-aged adults. N Engl J Med (1993) 328:1230–5 10.1056/NEJM199304293281704 8464434

[10] Botros N Concato J Mohsenin V Selim B Doctor K Yaggi HK Obstructive sleep apnea as a risk factor for type 2 diabetes. Am J Med (2009) 122:1122–7 10.1016/j.amjmed.2009.04.026 19958890 PMC2799991

[11] Punjabi NM Ahmed MM Polotsky VY Beamer BA O’Donnell CP Sleep-disordered breathing, glucose intolerance, and insulin resistance. Respir Physiol Neurobiol (2003) 136:167–78 10.1016/S1569-9048(03)00079-X 12853008

[12] Punjabi NM Caffo BS Goodwin JL Gottlieb DJ Newman AB O’Connor GT Sleep-disordered breathing and mortality: a prospective cohort study. PLoS Med (2009) 6:e1000132 10.1371/journal.pmed.1000132 19688045 PMC2722083

[13] Shahar E Whitney CW Redline S Lee ET Newman AB Nieto FJ Sleep-disordered breathing and cardiovascular disease: cross-sectional results of the sleep heart health study. Am J Respir Crit Care Med (2001) 163:19–25 10.1164/ajrccm.163.1.2001008 11208620

[14] Young T Finn L Peppard PE Szklo-Coxe M Austin D Nieto FJ Sleep disordered breathing and mortality: eighteen-year follow-up of the Wisconsin sleep cohort. Sleep (2008) 31:1071–8 18714778 PMC2542952

[15] Hendricks JC Kline LR Kovalski RJ O’Brien JA Morrison AR Pack AI The English bulldog: a natural model of sleep-disordered breathing. J Appl Physiol (1987) 63:1344–50 3693167 10.1152/jappl.1987.63.4.1344

[16] Lee MC Lee CH Hong SL Kim SW Lee WH Lim JY Establishment of a rabbit model of obstructive sleep apnea by paralyzing the genioglossus. JAMA Otolaryngol Head Neck Surg (2013) 139:834–40 10.1001/jamaoto.2013.4001 23949360

[17] Neuzeret PC Gormand F Reix P Parrot S Sastre JP Buda C A new animal model of obstructive sleep apnea responding to continuous positive airway pressure. Sleep (2011) 34:541–8 21461333 10.1093/sleep/34.4.541 PMC3065265

[18] Brennick MJ Pack AI Ko K Kim E Pickup S Maislin G Altered upper airway and soft tissue structures in the New Zealand obese mouse. Am J Respir Crit Care Med (2009) 179:158–69 10.1164/rccm.200809-1435OC 18996996 PMC2633061

[19] Nakano H Magalang UJ Lee SD Krasney JA Farkas GA Serotonergic modulation of ventilation and upper airway stability in obese Zucker rats. Am J Respir Crit Care Med (2001) 163:1191–7 10.1164/ajrccm.163.5.2004230 11316658

[20] Sood S Liu X Liu H Horner RL Genioglossus muscle activity and serotonergic modulation of hypoglossal motor output in obese Zucker rats. J Appl Physiol (2007) 102:2240–50 10.1152/japplphysiol.01229.2006 17332267

[21] Malhotra A Pillar G Fogel RB Beauregard J Edwards JK Slamowitz DI Genioglossal but not palatal muscle activity relates closely to pharyngeal pressure. Am J Respir Crit Care Med (2000) 162:1058–62 10.1164/ajrccm.162.3.9912067 10988130

[22] Oliven A Tov N Geitini L Steinfeld U Oliven R Schwartz AR Effect of genioglossus contraction on pharyngeal lumen and airflow in sleep apnoea patients. Eur Respir J (2007) 30:748–58 10.1183/09031936.00131106 17567673

[23] Saboisky JP Butler JE McKenzie DK Gorman RB Trinder JA White DP Neural drive to human genioglossus in obstructive sleep apnoea. J Physiol (Lond) (2007) 585:135–46 10.1113/jphysiol.2007.139584 17916615 PMC2375464

[24] McClung JR Goldberg SJ Functional anatomy of the hypoglossal innervated muscles of the rat tongue: a model for elongation and protrusion of the mammalian tongue. Anat Rec (2000) 260:378–86 10.1002/1097-0185(20001201)260:4<378::AID-AR70>3.0.CO;2-A 11074403

[25] Mu L Sanders I Human tongue neuroanatomy: nerve supply and motor endplates. Clin Anat (2010) 23:777–91 10.1002/ca.21011 20607833 PMC2955167

[26] Fuller DD Williams JS Janssen PL Fregosi RF Effect of co-activation of tongue protrudor and retractor muscles on tongue movements and pharyngeal airflow mechanics in the rat. J Physiol (Lond) (1999) 519:601–13 10.1111/j.1469-7793.1999.0601m.x 10457075 PMC2269504

[27] Rukhadze I Kalter J Kubin L Lingual muscle activity is increased in rats with surgically impaired geniohyoid muscle functions. Sleep (2010) 33:A20

[28] Lu JW Kubin L Electromyographic activity at the base and tip of the tongue across sleep-wake states in rats. Respir Physiol Neurobiol (2009) 167:307–15 10.1016/j.resp.2009.06.004 19539786 PMC2717949

[29] Rukhadze I Kamani H Kubin L Quantitative differences among EMG activities of muscles innervated by subpopulations of hypoglossal and upper spinal motoneurons during non-REM sleep – REM sleep transitions: a window on neural processes in the sleeping brain. Arch Ital Biol (2011) 149:499–515 10.4449/aib.v149i4.1385 22205596 PMC5222572

[30] Stettner GM Rukhadze I Mann GL Lei Y Kubin L Respiratory modulation of lingual muscle activity across sleep-wake states in rats. Respir Physiol Neurobiol (2013) 188:308–17 10.1016/j.resp.2013.05.033 23732510 PMC3778153

[31] Carley DW Trbovic S Radulovacki M Sleep apnea in normal and REM sleep-deprived normotensive Wistar-Kyoto and spontaneously hypertensive (SHR) rats. Physiol Behav (1996) 59:827–31 10.1016/0031-9384(95)02205-8 8778873

[32] Christon J Carley DW Monti D Radulovacki M Effects of inspired gas on sleep-related apnea in the rat. J Appl Physiol (1996) 80:2102–7 8806919 10.1152/jappl.1996.80.6.2102

[33] Davis EM O’Donnell CP Rodent models of sleep apnea. Respir Physiol Neurobiol (2013) 188:355–61 10.1016/j.resp.2013.05.022 23722067 PMC4010146

[34] Lu JW Mann GL Ross RJ Morrison AR Kubin L Differential effect of sleep-wake states on lingual and dorsal neck muscle activity in rats. Respir Physiol Neurobiol (2005) 147:191–203 10.1016/j.resp.2005.05.007 15964252

[35] Polotsky VY Wilson JA Haines AS Scharf MT Soutiere SE Tankersley CG The impact of insulin-dependent diabetes on ventilatory control in the mouse. Am J Respir Crit Care Med (2001) 163:624–32 10.1164/ajrccm.163.3.2007120 11254515

[36] Megirian D Hinrichsen CFL Sherrey JH Respiratory roles of genioglossus, sternothyroid, and sternohyoid muscles during sleep. Exp Neurol (1985) 90:118–28 10.1016/0014-4886(85)90045-7 4043287

[37] Hendricks JC Petrof BJ Panckeri K Pack AI Upper airway dilating muscle hyperactivity during non-rapid eye movement sleep in English bulldogs. Am Rev Respir Dis (1993) 148:185–94 10.1164/ajrccm/148.1.185 8317796

[38] Almeida FR Tsuiki S Hattori Y Takei Y Inoue Y Lowe AA Dose-dependent effects of mandibular protrusion on genioglossus activity in sleep apnoea. Eur Respir J (2011) 37:209–12 10.1183/09031936.00194809 21205716

[39] Winter WC Gampper T Gay SB Suratt PM Enlargement of the lateral pharyngeal fat pad space in pigs increases upper airway resistance. J Appl Physiol (1995) 79:726–31 8567510 10.1152/jappl.1995.79.3.726

[40] Gardinier ES Manal K Buchanan TS Snyder-Mackler L Altered loading in the injured knee after ACL rupture. J Orthop Res (2013) 31:458–64 10.1002/jor.22249 23097309 PMC3553294

[41] Brown EC Hudson AL Butler JE McKenzie DK Bilston LE Gandevia SC Single motor unit recordings in human geniohyoid reveal minimal respiratory activity during quiet breathing. J Appl Physiol (2011) 110:1054–9 10.1152/japplphysiol.00454.2010 21330618

[42] Stephenson R Horner RL The effect of time of day on apnoea index in sleeping rats. Respir Physiol Neurobiol (2006) 154:351–5 10.1016/j.resp.2006.02.007 16554190

[43] Mendelson WB Martin JV Perlis M Giesen H Wagner R Rapoport SI Periodic cessation of respiratory effort during sleep in adult rats. Physiol Behav (1988) 43:229–34 10.1016/0031-9384(88)90243-0 3212061

[44] Sato T Saito H Seto K Takatsuji H Sleep apneas and cardiac arrhythmias in freely moving rats. Am J Physiol (1990) 259:282–7 2386238 10.1152/ajpregu.1990.259.2.R282

[45] Guilleminault C Cummiskey J Progressive improvement of apnea index and ventilatory response to CO_2_ after tracheostomy in obstructive sleep apnea syndrome. Am Rev Respir Dis (1982) 126:14–20 6807156 10.1164/arrd.1982.126.1.14

